# Geosocial networking apps and disordered eating among queer Asian American men: a serial mediation model of racial oppression

**DOI:** 10.1186/s40337-026-01683-x

**Published:** 2026-06-17

**Authors:** Phúc Q. Phan, Thomas P. Le, Jason M. Nagata

**Affiliations:** 1 School of Counseling and Counseling Psychology, Arizona State University, Tempe, AZ USA; 2 Department of Psychology, Bryn Mawr College, Bryn Mawr, PA USA; 3 Department of Pediatrics, University of California, 550 16th Street, 4th Floor, Box 0503, San Francisco, CA USA

**Keywords:** Disordered eating, Sexual racism, Internalized racism, Queer Asian American men, Geosocial networking apps

## Abstract

**Background:**

Queer Asian American men face pervasive experiences of racism in romantic and sexual contexts (i.e., sexual racism), which may heighten their risk of developing disordered eating concerns. Moreover, geosocial networking apps (GNAs) used by queer men are a pertinent risk factor for disordered eating attitudes and behaviors. Nevertheless, no studies have examined the links between GNAs, racially relevant factors, and disordered eating among queer Asian American men. Hence, grounded in intersectionality theory and the racially expanded model of objectification theory, the present study investigates the associations between GNA intensity and GNA sex-seeking with disordered eating through sexual racism and internalized racism sequentially.

**Methods:**

Participants were recruited through Prolific. Our final sample consisted of 265 queer Asian American men whose ages ranged from 18 to 65 (*M* = 28.42, *SD* = 8.32). All analyses were conducted through SPSS (v.29).

**Results:**

Serial mediation analyses indicated that GNA intensity was indirectly associated with greater disordered eating through sexual racism and internalized racism sequentially (*B* = 0.028, *SE* = 0.013, 95% CI [0.008, 0.058]). Similarly, GNA sex-seeking was indirectly associated with greater disordered eating through sexual racism and internalized racism sequentially (*B* = 0.061, *SE* = 0.026, 95% CI [0.018, 0.118]).

**Conclusions:**

As queer Asian American men engage with GNAs intensively and for sex-seeking purposes, it is possible that they may face sexual racism and subsequently experience internalized racism, which consequentially is associated with greater disordered eating. Social justice-informed implications are discussed. For instance, GNAs may consider implementing educational advertisements to inform users of the pervasiveness of sexual racism and antiracist policies that integrate features that flag and remove profiles or interactions with racist language.

## Abstract (plain language summary)

The disordered eating literature has scarcely centered the experiences of queer Asian American men, who face a heightened risk for disordered eating attitudes and behaviors due to intersectional axes of oppression. Specifically, experiences of racism in romantic and sexual contexts, otherwise known as sexual racism, have been linked to disordered eating among this community. In this sample of 265 queer Asian American men, we found that as queer Asian American men use online dating/hookup apps intensively and for sex-seeking purposes, they may face sexual racism and, subsequently, experience internalized racism, which increases their risk for disordered eating. Our study highlights online dating/hookup platforms as a potential factor associated with disordered eating among queer Asian American men through sexual racism and internalized racism. Disordered eating interventions may integrate social justice-informed frameworks that address racial oppression to reduce disordered eating attitudes and behaviors among queer Asian American men. Furthermore, online dating/hookup apps may implement prevention efforts to preclude the onset of disordered eating by applying anti-racist policies that target sexual racism experiences, as well as disseminating psychoeducational advertisements that provide consciousness-raising strategies to combat internalized racism.

## Introduction

The disordered eating body of research has expanded its emphasis on White, female, college-aged samples to highlight the experiences of queer men, who face a disproportionately high risk for disordered eating symptoms compared to their heterosexual counterparts [1]. According to the minority stress theory [2, 3], queer men’s vulnerability to disordered eating symptoms may be explained by their experiences of societal stigma, prejudice, and discrimination related to their sexual minority status [4, 5]. However, limited studies have specifically examined the experiences of queer men of color. This oversight omits a vulnerable community in the literature, considering how queer men of color experience greater disordered eating symptoms compared to queer White men due to intersecting axes of oppression [6–10], particularly related to race and ethnicity [6, 8, 11–13].

Sociocultural theories of eating disorders and body image provide a critical understanding of how systems of oppression may drive disordered eating attitudes and behaviors among queer men of color [14]. For example, the tripartite influence model highlights the influence of parents, peers, and media on body image and eating disturbances through comparison and internalization [15]. One important contribution to this body of research is the culturally expanded model of objectification theory [16] and, by extension, the racially expanded framework [17], where racism is conceptualized as an objectifying process that can lead to the development of eating pathology among racial minorities. Objectification theory was first conceptualized to highlight how women are frequently viewed and treated as merely physical and sexual objects by men in society [18]. As women are confronted with these pervasive sexually objectifying messages, they may internalize the male gaze and adopt objectifying beliefs and behaviors that put them at greater risk for disordered eating [19]. Applying these models to queer Asian American men, this community may experience a pertinently high risk of developing disordered eating concerns, considering how they face pervasive racial discrimination within the queer community [20, 21]. Specifically, research suggests that exposure to emasculating stereotypes of Asian American men as effeminate and undesirable may render this population more vulnerable to negative muscularity-related attitudes [22–24], putting them at greater risk for disordered eating symptoms compared to other racial groups [22, 25]. For instance, gendered racism is associated with greater muscularity-oriented disordered eating symptoms [26], and perpetual foreigner racism is associated with a higher drive for muscularity among this community [27].

Nevertheless, the few studies that examined Asian American men’s eating pathology have primarily focused on heterosexual samples, with only one known study highlighting queer Asian American men specifically [28]. Considering how mortality rates have elevated among individuals with eating disorders in recent years [29], and how social justice-informed research underscores the importance of breaking down the disordered eating literature to focus on queer men of color [30, 31], more research is needed to identify factors associated with eating pathology among queer Asian American men.

### Geosocial networking app use

Geosocial networking apps (GNAs; e.g., Grindr, Tinder) are a pertinent risk factor for negative body image and disordered eating among queer men [32, 33]. While these online communities can provide several benefits (e.g., safe self-expression, community building), greater usage of these platforms is also associated with various negative health outcomes, including lower self-esteem [34], increased sexual risk behaviors [35–37], and engagement in unhealthy weight control and muscle-enhancing behaviors [33]. GNA use as a risk factor for disordered eating may be particularly salient among queer men of color, given that this community frequently faces intersectional stigma tied to White supremacist body standards and gendered racial stereotypes in these online platforms [20, 34]. For instance, queer men of color in a 2024 qualitative study identify online dating as a source of objectification that engenders their disordered eating symptoms, with one queer Asian American man reporting how exposure to the glorification of Whiteness in online dating apps negatively influences his body image and relationship with eating [38]. Similarly, a recent quantitative study highlights that among Chinese young gay men, gay dating app usage is linked with both thinness and muscularity-oriented disordered eating [39]. Furthermore, queer Asian men frequently face anti-Asian sentiments on GNAs, including exclusionary languages such as “no Asians” or emasculating messages that denigrate Asian men’s desirability [40]. In fact, a large proportion of queer Asian men (93%) in a recent quantitative study reported facing sexual racism on GNAs [41]. Given that many queer Asian American men use virtual platforms to search for dating and sexual partners [42] and those who use GNAs intensively are more likely to experience muscle dysmorphia [43], more efforts are needed to understand how usage of these platforms may influence disordered eating among this community.

In the present study, we conceptualize GNA use through two distinct dimensions: sex-seeking (GNA sex-seeking) and intensity of use (GNA intensity), given that the motivations behind why queer Asian American men engage with these platforms may vary. First, the present study examines the extent to which users seek sex on GNAs, a common motivation among queer men in general [44] and queer Asian American men in particular [42]. For example, 63.6% of men in a 2014 study reported having used an app to meet a sexual partner [45]. At the same time, it is important to recognize that queer men may also engage with GNAs for other purposes, such as seeking social support or romantic partners [46]. Thus, we also investigate GNA intensity, conceptualized as the level of emotional connection users have with these virtual communities and the extent to which these online platforms are embedded in their personal lives via temporal and mental investment [47]. By examining sex-seeking and intensity of use as separate variables, the present study aims to provide a more nuanced understanding of how different uses of GNAs influence disordered eating among queer Asian American men.

### Sexual racism

One particular stressor that may explain why GNA intensity and sex-seeking may be associated with disordered eating among queer Asian American men is racial discrimination in romantic and sexual contexts, a phenomenon known as sexual racism [20, 21, 30]. Here, intersectionality theory [48], originally coined to conceptualize Black women’s experience with multidimensional systems of oppression due to race and gender, serves as an important framework in understanding queer Asian American men’s multilayered identities. Situated at the crossroads of masculinity, gender, and sexuality, queer Asian American men are often objectified and emasculated by harmful stereotypes that portray them as effeminate, small, unattractive, and ultimately undesirable, relegating them to the bottom of the racial hierarchy of attractiveness within the queer community [21, 49, 50]. Here, it is important to note that this racial hierarchy in the queer community is not based on the ontological reality of White men being superior to men of color, but it is rather socially constructed through systemic and historical forces of White supremacy in the United States [51]. Research examining this unique dimension of racial oppression among queer Asian American men is critical, considering how it is associated with a myriad of negative health outcomes, including feelings of isolation and marginalization [50], sexual risk behaviors [21], depression and anxiety [36, 52], and muscle dysmorphia [53].

We hypothesize that sexual racism will serve as the first serial mediator in explaining the associations between GNA intensity and sex-seeking with disordered eating. As discussed earlier, when queer Asian American men engage with GNAs, they frequently contend with racist stereotypes that denigrate their sexual desirability and fetishize their physical appearance [21, 34, 49]. These objectifying and dehumanizing messages reinforce a racial hierarchy of desire within the queer male community where queer White men are glorified at the top while queer Asian American men are relegated to the bottom [30, 50]. Building upon the racially expanded model of objectification theory among Asian American college women [17], experiences of sexual racism may drive queer Asian American men to engage in disordered eating behaviors to negotiate their perceived lack of masculinity and modify their bodies to conform to White supremacist beauty standards [43, 53]. Our hypothesized finding is built upon a 2023 study among queer Asian American men [28], in which sexual racism was found to be associated with greater disordered eating; nevertheless, this study did not examine sexual racism within the context of GNAs. Thus, the present study seeks to advance the literature by investigating how experiencing sexual racism from GNAs may prompt disordered eating behaviors and attitudes among queer Asian American men.

### Internalized racism

As queer Asian American men navigate instances of sexual racism from GNAs, they may adopt inferiorized and racist attitudes and beliefs toward their racial identity, a proximal form of racial oppression known as internalized racism [54]. Specifically, internalized racism may drive queer Asian American men to devalue their racial identity and heritage and develop a negative self-concept [55]. This destructive form of racism is associated with a host of negative mental and behavioral health outcomes among Asian American adults (e.g., depressive symptoms, lower collective racial self-esteem, disordered eating) [54, 56], as well as queer Asian American men (e.g., disordered eating, muscle dysmorphia) [28, 43].

We hypothesize that internalized racism will serve as the second serial mediator in explaining the associations between GNA intensity and sex-seeking with disordered eating. Specifically, as queer Asian American men face sexual racism from engaging with GNAs intensively and for sex-seeking purposes, they may experience internalized racism, which, in turn, increases their risk for disordered eating. Similar to how a 2023 study found that emotional eating mediated the association between internalized racism and disordered eating [28], queer Asian American men who experience internalized racism from exposure to sexual racism may engage in disordered eating as a maladaptive coping mechanism to regulate feelings of distress. For instance, one queer Asian American man in a 2024 qualitative study reported using food as a way to cope with the intense anxiety and emotional distress stemming from facing rejection in the queer community and self-hatred toward his perceived undesirable body [38]. It is also possible that queer Asian American men with internalized racism may strive to fulfill White hegemonic masculinity ideals by engaging in disordered eating as a compensatory behavior to gain greater muscularity and to be perceived as more masculine [24, 27, 43].

### The present study

While disordered eating research among Asian American individuals has been growing among Asian American women [17, 56–58] and Asian American men [26], the extension of this work to queer Asian American men remains deeply underdeveloped. Hence, grounded in intersectionality theory [48] and the racially expanded model of objectification theory [17], the present study investigates the associations between GNA intensity and sex-seeking with disordered eating through sexual racism and internalized racism sequentially. Specifically, we hypothesize that the more queer Asian American men engage with GNAs intensively and for sex-seeking purposes, the more probable they are to face sexual racism and subsequently experience internalized racism, which consequentially will be associated with greater disordered eating symptoms.

## Methods

### Participants and procedures

The study received approval from the Institutional Review Board before data collection. All procedures for the current study were executed online. To participate in the study, participants had to meet three inclusion criteria: (1) be 18 years old or older, (2) identify as a queer Asian American man, and (3) live in the United States (U.S.). Recruitment was completed remotely through Prolific, an online recruitment platform that utilizes a double opt-in process, such that after participants share their demographic information with the platform, they are invited to partake in studies for which they qualify. Prolific also automatically prevents participants from making multiple accounts and removes duplicate responses. Furthermore, an analysis of online data recruitment platforms and panels provided evidence that Prolific’s data quality and validity were better than those of other platforms (i.e., Amazon Mechanical Turk, CloudResearch) [59].

Participants who met the three inclusion criteria completed informed consent and then filled out the remainder of the survey, which included a series of questionnaires related to the study’s demographic items, variables of interest, and two validity check items (e.g., “To indicate that you are paying attention, please select ‘Agree’ for this item”). Participants were paid about $16.65 per hour.

The present study’s final sample consisted of 265 queer Asian American men who completed the survey in 2024. A total of 326 participants initially accessed the survey. Of those 326 individuals, 39 only completed the informed consent form and screening items, six only completed the first questionnaire about geosocial networking apps, and 16 did not identify as Asian (e.g., they identified as either Black or White). We retained participants who only missed one of two validity checks, and no participants missed both. Thus, our final sample comprised 265 queer Asian American men whose ages ranged from 18 to 65 (*M* = 28.42, *SD* = 8.32). The majority of participants were born in the U.S. (*n* = 219 born in the U.S.; *n* = 39 born outside of the U.S.). Table 1 provides comprehensive information about participants’ demographics.

**Table 1:** Sample demographics

Variable	Frequency	Percent	Valid Percent
Ethnicity			
Chinese	34	12.8	13.1
Japanese	16	6.0	6.2
Korean	20	7.5	7.7
Filipino	44	16.6	16.9
Vietnamese	29	10.9	11.2
Taiwanese	5	1.9	1.9
Indian	17	6.4	6.5
Native Hawaiian or Pacific Islander	4	1.5	1.5
Multiracial and/or Multiethnic	68	25.7	26.2
Not listed above	12	4.5	4.6
Born in the United States			
Yes	219	82.6	84.2
No	39	14.7	15.0
Unsure	2	0.8	0.8
Gender			
Man	248	93.6	95.4
Genderfluid	5	1.9	1.9
Gender non-conforming	2	0.8	0.8
Not listed above	5	1.9	1.9
Transgender			
Yes	42	15.8	16.2
No	218	82.3	83.8
Sexual orientation			
Bisexual	136	51.3	52.3
Gay	91	34.3	35.0
Heterosexual	3	1.1	1.2
Questioning	2	0.8	0.8
Queer	19	7.2	7.3
Asexual	3	1.1	1.2
Not listed above	6	2.3	2.3
Generational status			
1st Generation	36	13.6	13.8
1.5 Generation	13	4.9	5.0
2nd Generation	162	61.1	62.3
3rd Generation and beyond	44	16.6	16.9
Adoptee	1	0.4	0.4
Not listed above	4	1.5	1.5
Educational Attainment			
High school diploma or less	21	7.9	8.1
Some college	83	31.3	31.9
College degree	113	42.6	43.5
Professional or graduate degree	42	15.8	16.2
Household income			
Under $10,000	13	4.9	5.0
$10,000-$14,999	7	2.6	2.7
$15,000-$24,999	9	3.4	3.5
$25,000-$34,999	26	9.8	10.0
$35,000-$49,999	26	9.8	10.0
$50,000-$74,999	58	21.9	22.3
$75,000-$99,999	42	15.8	16.2
$100,000-$199,999	46	17.4	17.7
$200,000 or greater	22	8.3	8.5
Unsure	11	4.2	4.2

Given the large number of ethnic identifications in this sample, we opted to provide the most common categories for clarity of presentation. 1.1% or fewer participants identified with the following ethnicities: Cambodian, Thai, Hmong, Laotian, Bangladeshi, Indonesian, and Malaysian

### Measures

#### Demographics

Participants responded to a variety of demographic items including gender, age, generational status, sexual orientation, educational attainment, ethnicity, race, and whether participants were born inside or outside of the U.S. Following guidelines provided by the American Psychological Association [60], we used the label “queer” as an umbrella term to refer to Asian American men who identify as a sexual minority (i.e., gay, questioning, bisexual, asexual). Furthermore, men may also identify as queer and being attracted to men without having actually engaged in sexual intercourse with another man, which is another reason we used the term “queer” specifically.

### Geosocial networking app use intensity

The adapted version of the Facebook Intensity Scale [47] derived from an analysis of Facebook and social capital [61] was used to assess geosocial networking app use intensity (GNA intensity). Participants rated six attitudinal items (e.g., “I feel out of touch when I haven’t logged onto sexual minority men geosocial networking apps for a while”) on a 7-point Likert scale ranging from 1 (*Disagree strongly*) to 7 (*Agree strongly*). They also rated one behavioral statement (“In the past month, on average, approximately how many minutes per day did you spend on this app?”) on a 4-point ordinal scale (10 min or less per day, 10–30 min per day, 31–60 min per day, and more than 60 min per day). All items were summed to compute a composite score where higher scores indicated greater app use intensity. The measure has demonstrated adequate reliability among a sample of queer Asian American men (Cronbach’s alpha = 0.93) [43]. Cronbach’s alpha was 0.90 in the present study.

### Geosocial networking app use sex-seeking

The Motive of Sex-Seeking measure [47] was used to assess the extent to which participants engaged in a geosocial networking app to seek sex (GNA sex-seeking). Participants responded to three items (e.g., “I use sexual minority men geosocial networking apps to arrange a time or place to hook up”) on a 7-point Likert scale ranging from 1 (*Never*) to 7 (*Always*). Items were summed to calculate a composite score where higher scores indicated greater GNA sex-seeking. The measure has yielded adequate reliability among queer Asian American men (Cronbach’s alpha = 0.93) [43]. Cronbach’s alpha in the present study was 0.93.

### Sexual racism

The Experienced Sexual Racism Items [62] derived from the Sexual Racism Scale [63] were used to assess participants’ experiences with racial discrimination in romantic and/or sexual contexts. Participants responded to five questions about the frequency of experiences of sexual racism they encountered either in person or online (e.g., “People have approached me for sex or dating only because of my race/ethnicity, they don’t care about my personal characteristics”) and one item about whether they felt stressed during these experiences (e.g., “When I have experienced any discrimination on mobile apps/Internet/real life because of my race/ethnicity, I have been stressed about it”). All items were rated on a 4-point Likert scale ranging from 1 (*Never*) to 4 (*Most of the time*), with higher total scores indicating a greater frequency of experiencing sexual racism. The scale has demonstrated adequate reliability among samples of queer Asian American men specifically (Cronbach’s alpha = 0.87; Cronbach’s alpha = 0.89) [52, 53]. Cronbach’s alpha for the present study was 0.87.

### Internalized racism

The Internalized Racism in Asian Americans Scale (IRAAS) [54] was used to assess participants’ negative attitudes toward their Asian racial identity. Participants rated 14 items on a 6-point Likert-type scale ranging from 1 *(Strongly Disagree)* to 6 *(Strongly Agree)* in three factors: (1) Self-Negativity (e.g., “It’s unfair that I was born Asian”), (2) Weakness Stereotypes (e.g., “Asian men are more feminine than non-Asian men”), and (3) Appearance Bias (e.g., “Many Asians would be more physically attractive if they had surgery to look more White”). Items across the three subscales were summed to generate a composite score, such that higher scores denoted greater levels of internalized racism. The measure development study reported evidence of convergent validity, predictive validity, and high reliability (Cronbach’s alpha = 0.93) [54]. The IRAAS has also demonstrated adequate reliability among samples of queer Asian American men (Cronbach’s alpha = 0.87; Cronbach’s alpha = 0.93) [43, 64]. Cronbach’s alpha for the present study was 0.93.

### Disordered eating

The seven-item version of the Eating Disorder Examination-Questionnaire (EDE-Q7) [65], derived from an evaluation of eating disorder assessments [66], was used to assess participants’ disordered eating symptoms in the past four weeks (28 days). Participants responded to seven items in three factors: (1) Dietary Restraint (e.g., “Have you been deliberately trying to limit the amount of food you eat to influence your shape or weight, whether or not you have succeeded?”), (2) Shape/Weight Overvaluation (e.g., “Has your weight influenced how you think about (judge) yourself as a person?”), and (3) Body Dissatisfaction (e.g., “How dissatisfied have you been with your weight”). Items in the Dietary Restraint subscale were rated on a 7-point Likert scale ranging from 1 (*No days*) to 7 *(Every day*), and items in the Shape/Weight Overvaluation and Body Dissatisfaction subscales were rated on a 7-point Likert scale ranging from 1 (*Not at all*) to 7 *(Markedly*). A composite score was generated by summing items across the three factors, such that greater total scores denoted higher disordered eating symptoms. The EDE-Q7 has demonstrated evidence of measurement invariance and adequate internal consistency among samples of cisgender gay men (Cronbach’s alpha = 0.85) [67] and transgender men (Cronbach’s alpha = 0.82) [68]. Cronbach’s alpha was 0.91 among the present study’s sample of queer Asian American men.

### Data analytic plan

The data analytic plan was created before conducting data analysis. SPSS (v.29) was used to conduct analyses, and alpha values *p* < .05 were regarded as statistically significant. Multivariate outliers and multicollinearity were investigated. Kurtosis and skewness were also investigated to assess the data’s normality. After examining bivariate correlations between the study’s primary variables and demographic variables, we utilized a linear regression analysis to examine whether GNA intensity and sex-seeking were associated with disordered eating. We then ran serial mediation analyses utilizing the PROCESS macro in SPSS to assess the serial indirect effects of sexual racism and internalized racism.

## Results

### Data screening and preparation

Skewness for the study’s primary variables of interest ranged from 0.04 to 0.64, and kurtosis ranged from − 1.14 to − 0.24, both of which fell within the acceptable range [69]. We conducted a Little’s Missing Completely at Random (MCAR) analysis and found a nonsignificant chi-square statistic χ2 (4) = 1.10, *p* = .90, indicating that data were missing at random. Specifically, five individuals did not complete the eating disorder questionnaire; all other participants responded to all measures fully. Thus, as modeled by Parent’s [70] guidelines on addressing missing data, we utilized pairwise deletion, such that available data were utilized for analyses and missing data were removed solely for analyses that included those missing data points.

### Correlation analyses

Table 2 contains correlations and descriptive statistics for our primary variables of interest. Both GNA sex-seeking and GNA intensity were positively and significantly correlated with sexual racism and internalized racism. Additionally, both sexual racism and internalized racism were positively and significantly correlated with disordered eating. None of the demographic variables were correlated with disordered eating. Furthermore, given past research indicating that transgender individuals are at greater risk of disordered eating than their cisgender counterparts [71], an independent samples *t*-test was conducted to compare group means of disordered eating between transgender and cisgender queer Asian American men. Results indicated that there was no significant difference in disordered eating between transgender and cisgender participants, *t*(258) = 0.47, *p* = .64. Thus, as done in past research with queer Asian American men [43], we did not include demographic variables as covariates in subsequent analyses.

**Table 2:** Correlations matrix of variables of interest

Variable	Mean (SD)	1	2	3	4	5	6	7	8	9
1. GNA intensity	20.89 (*9.76*)	–								
2. GNA sex-seeking	9.64 (*4.98*)	0.68**	–							
3. Sexual racism	11.83 (*3.95*)	0.40**	0.42**	–						
4. Internalized racism	32.75 (*13.89*)	0.24**	0.21**	0.38**	–					
5. Disordered eating	26.88 (*12.05*)	0.06	0.07	0.19**	0.22**	–				
6. Age	28.43 (*8.32*)	0.19**	0.20**	0.15*	0.14*	0.03	–			
7. Educational attainment	3.70 (.*88*)	0.24**	0.18**	0.16**	0.19**	− 0.06	0.34**	–		
8. Income	6.18 (*2.19)*	− 0.11	− 0.09	− 0.08	− 0.16**	− 0.03	− 0.03	0.19**	–	
9. Nativity	1.17 (.*39)*	0.08	0.05	− 0.02	− 0.03	− 0.06	0.06	0.06	0.07	–

GNA = Geosocial networking app

*p < *.*05, **p < *.*01

### Regression analyses

We first executed a multiple regression analysis that examined the associations of GNA sex-seeking and GNA intensity with disordered eating. Before conducting this analysis, we calculated Cook’s distance (D) to investigate if multivariate outliers influenced our data to an undue extent. Since all cases had a Cook’s D < 1, we concluded that multivariate outliers did not unduly influence our analysis because D > 1 is commonly conceptualized as an indication for outliers [72]. Moreover, all variance inflation factors were less than 2, and all tolerance coefficients were greater than 0.20, indicating no significant multicollinearity.

In the linear regression analysis, we entered GNA intensity and GNA sex-seeking as the independent variables with disordered eating as the dependent variable. This model accounted for 0.5% of the variance in disordered eating, a non-significant amount (*F*(2, 257) = 0.647, *p* = .524). Neither GNA intensity (β = 0.03, *B* = 0.04, *t* = 0.385, *p* = .700) nor GNA sex-seeking (β = 0.05, *B* = 0.11, *t* = 0.530, *p* = .596) were significantly associated with disordered eating.

### Mediation analyses

To examine whether sexual racism and internalized racism sequentially mediated the associations between GNA intensity and sex-seeking with disordered eating, we conducted serial mediation analyses with PROCESS Model 6 in SPSS [73]. As per recommended practices [73], we utilized the bias-corrected bootstrapping procedure to generate 10,000 bootstrap samples to construct the 95% CIs of indirect effects. We investigated two models, the first with GNA intensity as the independent variable and the second with GNA sex-seeking as the independent variable. For both models, sexual racism was the first mediator and internalized racism was the second mediator, and disordered eating was the dependent variable. If the confidence interval does not include zero, the indirect effect is significant [73, 74]. Table 3 displays statistics for both serial mediation models.

**Table 3:** Direct and indirect effects of GNA intensity and sex-seeking on disordered eating through sexual racism and internalized racism

IV	Mediator(s)	DV	B	SE	β	95% bootstrap CI
Model 1						
Total direct effect						
GNA intensity		DO	− 0.04	0.08	− 0.03	[-0.201, 0.121]
Total indirect effect						
GNA intensity		DO	0.12	0.04	0.10	[0.045, 0.199]
Specific indirect effect						
GNA intensity	→ SR →	DO	0.07	0.03	0.05	[-0.001, 0.143]
GNA intensity	→ IR →	DO	0.02	0.02	0.02	[-0.004, 0.063]
GNA intensity	→ SR → IR →	DO	0.03	0.01	0.02	[0.008, 0.058]
Model 2						
Total direct effect						
GNA sex-seeking		DO	− 0.06	0.16	− 0.02	[-0.377, 0.258]
Total indirect effect						
GNA sex-seeking		DO	0.22	0.07	0.09	[0.078, 0.370]
Specific indirect effect						
GNA sex-seeking	→ SR →	DO	0.14	0.07	0.06	[-0.006, 0.280]
GNA sex-seeking	→ IR →	DO	0.02	0.03	0.01	[-0.025, 0.099]
GNA sex-seeking	→ SR → IR →	DO	0.06	0.03	0.03	[0.018, 0.118]

IV = independent variable; DV = dependent variable; SE = standard error; CI = confidence interval; GNA = geosocial networking app; SR = sexual racism; IR = internalized racism; DO = disordered eating

**Fig. 1:**
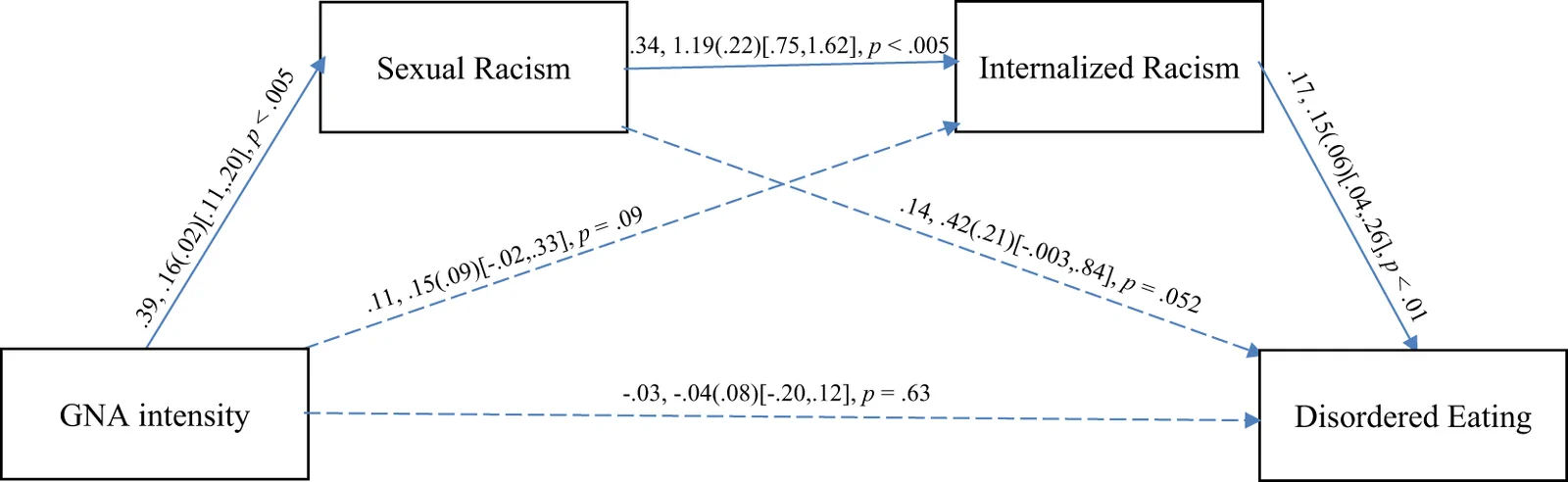
A serial mediation model depicting the indirect effects of GNA intensity on disordered eating via sexual racism and internalized racism, in standardized, unstandardized serial. beta estimate(standard error)[95% confidence interval]. Nonsignificant pathways are presented with dotted lines. Total indirect effect = 0.12, (SE = 0.04, 95% CI [0.05, 0.20])*

For our first model, the serial indirect effect of GNA intensity on disordered eating through sexual racism and internalized racism was significant (β *=* 0.023, *B* = 0.028, *SE* = 0.013, 95% CI [0.008, 0.058]). The direct effect of GNA intensity on disordered eating was not significant when introducing this serial indirect effect (β *=* − 0.032, *B* = − 0.040, *SE* = 0.082, *p* = .625), indicating full mediation. Thus, greater GNA intensity was associated with greater sexual racism, which was then associated with higher internalized racism; higher internalized racism was then associated with greater disordered eating. In fact, this pathway (GNA intensity → sexual racism → internalized racism → disordered eating) was the only significant indirect pathway in the model. Specifically, the individual indirect pathways from GNA intensity to disordered eating through sexual racism (β *=* 0.054, *B* = 0.07, *SE* = 0.04, 95% CI [-0.001, 0.143]) and, separately, internalized racism (β *=* 0.019, *B* = 0.02, *SE* = 0.02, 95% CI [-0.004, 0.063]) were not significant. This model accounted for 6.1% of the variance in disordered eating. These results suggest that (a) neither sexual racism nor internalized racism independently mediate the link between GNA intensity and disordered eating, and (b) both sexual racism and internalized racism collectively mediate the link between GNA intensity and disordered eating. Figure 1 displays this model.

**Fig. 2:**
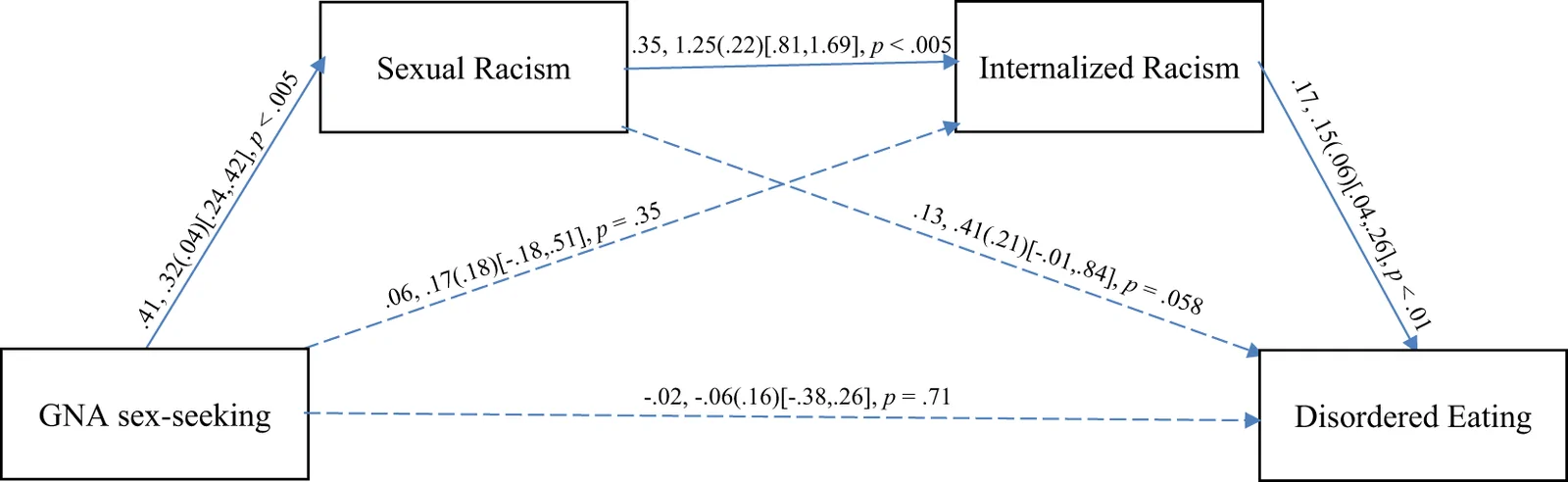
A serial mediation model depicting the indirect effects of GNA sex-seeking on disordered eating via sexual racism and internalized racism, in standardized, unstandardized serial. beta estimate(standard error)[95% confidence interval]. Nonsignificant pathways are presented with dotted lines. Total indirect effect = 0.22, (SE = 0.07, 95% CI [0.08, 0.37])*

For our second model, the serial indirect effect of GNA sex-seeking on disordered eating through sexual racism and internalized racism was significant (β *=* 0.025, *B* = 0.061, *SE* = 0.026, 95% CI [0.018, 0.118]). The direct effect of GNA sex-seeking on disordered eating was not significant when introducing this serial indirect effect (β *=* − 0.025, *B* = − 0.060, *SE* = 0.161, *p* = .709), indicating full mediation. Thus, greater GNA sex-seeking was associated with greater sexual racism, which was then associated with higher internalized racism; higher internalized racism was then associated with greater disordered eating. In fact, this pathway (GNA sex-seeking → sexual racism → internalized racism → disordered eating) was the only significant indirect pathway in the model. Specifically, the individual indirect pathways from GNA sex-seeking to disordered eating through sexual racism (β *=* 0.056, *B* = 0.14, *SE* = 0.07, 95% CI [-0.006, 0.280]) and, separately, internalized racism (β *=* 0.010, *B* = 0.02, *SE* = 0.03, 95% CI [-0.025, 0.099]) were not significant. This model accounted for 6.1% of the variance in disordered eating. These results suggest that (a) neither sexual racism nor internalized racism independently mediate the link between GNA sex-seeking and disordered eating, and (b) sexual racism and internalized racism collectively mediate the link between GNA sex-seeking and disordered eating. Figure 2 displays this model.

### Post-hoc reverse mediations

Considering the cross-sectional nature of the study, we utilized the same serial mediation procedures to conduct reverse serial mediation models with internalized racism as the first mediator and sexual racism as the second mediator. We found that neither of the reverse serial mediation models were significant, including the model with GNA intensity as the independent variable (β *=* 0.010, *B* = 0.012, *SE* = 0.008, 95% CI [-0.0003, 0.030]) and the model with GNA sex-seeking as the independent variable (β *=* 0.008, *B* = 0.020, *SE* = 0.013, 95% CI [-0.001, 0.054]).

## Discussion

In response to the call to integrate an intersectional lens into disordered eating research [1, 7], the present study is the first to examine the associations between GNA intensity and GNA sex-seeking with disordered eating through sexual racism and internalized racism sequentially. Aligning with our hypothesis, GNA intensity was indirectly associated with greater disordered eating through sexual racism and internalized racism. Similarly, GNA sex-seeking was indirectly associated with greater disordered eating through sexual racism and internalized racism. In other words, as queer Asian American men engage with GNAs intensively and for sex-seeking purposes, they may face sexual racism and subsequently experience internalized racism, which consequentially is associated with greater disordered eating.

The present study advances the extant literature on the role of GNAs as being associated with disordered eating among queer men [32, 33] by extending these findings to queer Asian American men specifically. The present study also provides a nuanced understanding of the associations between GNA intensity and GNA sex-seeking with disordered eating by underscoring the underlying mechanisms of sexual racism and internalized racism. Furthermore, our findings advance growing research on the link between GNAs and queer Asian American men’s well-being by highlighting that, in addition to muscle dysmorphia [43] and sexual risk behaviors [37], disordered eating is also a pertinent mental health concern. Additionally, our findings on the effects of sexual racism and internalized racism align with Le et al. [28] and provide further evidence to substantiate the racially expanded model of objectification theory [17]. Our findings also contribute to the sexual racism literature among queer Asian American men by indicating that disordered eating is also a notable mental health concern in addition to sexual risk behaviors [21], depression and anxiety [36, 52], and muscle dysmorphia [53]. Lastly, the present study expands the limited yet growing body of research on internalized racism and well-being among queer Asian American men [28, 53, 75].

In support of our hypotheses, both GNA intensity and GNA sex-seeking were indirectly associated with greater disordered eating through sexual racism and internalized racism sequentially. Our findings suggest that queer Asian American men may encounter sexual racism and subsequently experience internalized racism regardless of whether they are using GNAs intensively or to seek sex, indicating the pervasiveness of sexual racism in these online communities. Furthermore, it is important to point out that GNA intensity and GNA sex-seeking were not directly associated with disordered eating, demonstrating the powerful role that sexual racism and internalized racism play as factors associated with disordered eating among queer Asian American men. This lack of direct effects may be explained by considering how it is possible that the intensive usage of GNAs and the motive of sex-seeking alone are not inherently harmful for queer Asian American men. In fact, studies have documented that using GNAs may carry several benefits, such as allowing queer Asian American men to build affirming communities with other queer men of color [76] and cultivating higher self-esteem and life satisfaction [77]. Hence, our findings suggest that it is when queer Asian American men face sexual racism in these virtual communities that they may begin facing a heightened risk for disordered eating. Furthermore, integrating an outcomes balance perspective of risk [78], queer Asian American men in the present study may engage in various positive body image practices, such as body appreciation [79], that protect them from disordered eating symptoms when using GNAs, rendering the direct links between GNA intensity and sex-seeking with disordered eating non-significant. The paths from GNA intensity and GNA sex-seeking to sexual racism may be explained by considering how previous research has demonstrated that queer Asian American men frequently face emasculating messages that reduce them to stereotypes of femininity, submissiveness, and unattractiveness in romantic and sexual contexts [34, 49, 50]. Subsequently, as queer Asian American men navigate these denigrating messages, they may internalize objectifying and racist ideologies about their racial identity, thus developing internalized racism [30, 50, 75].

Among queer Asian American men who experience internalized racism, they may consequently develop disordered eating attitudes and behaviors for various reasons. First, given the emasculating nature of stereotypes targeting queer Asian American men [50], those with internalized racism may engage in disordered eating as an over-compensatory behavior to gain more muscularity to negotiate their perceived lack of masculinity [24, 27]. Furthermore, considering how masculinity often equates with Whiteness and desirability in the queer male community, queer Asian American men with internalized racism may perform maladaptive eating patterns to modify their bodies to fulfill White hegemonic masculinity ideals [43, 53]. It is also possible that queer Asian American men may develop disordered eating in efforts to regulate anxiety and emotional distress stemming from experiences of sexual racism and self-hatred toward their bodies [28, 38]. Lastly, queer Asian American men may also engage in disordered eating as a form of self-protection, such that if their bodies conform to White supremacist standards of beauty, they may be more likely to evade experiences of sexual racism [17].

Furthermore, it is important to note that our post hoc analyses indicated that the serial mediation pathway was no longer significant when the mediators were reversed, in which sexual racism was the second mediator and internalized racism was the first. The absence of significant indirect effects in this reverse mediation model provides further evidence that this pathway is not bidirectional and underscores the potential role of GNAs as a factor associated with disordered eating, operating through sexual racism and internalized racism, among queer Asian American men.

Lastly, it would be remiss not to point out that only a small proportion of variance in disordered eating was explained in both models. This suggests that queer Asian American men’s engagement in disordered eating may be shaped by a range of sociocultural factors in addition to GNAs, sexual racism, and internalized racism, particularly given their intersecting marginalized identities. For example, alongside experiences of sexual racism and internalized racism, queer Asian American men frequently encounter heterosexism and cisgenderism within broader society and within Asian communities [80, 81]. These systems of oppression have been linked to disordered eating symptoms among queer populations [4]. Moreover, in addition to GNAs, queer Asian American men may encounter discriminatory messages that demean their racial identity and physical features across non-dating-specific media platforms. For instance, a 2021 qualitative analysis of posts on the social media platform X (formerly Twitter) found that queer Asian American men are frequently stereotyped as weak and sexually passive [82]. This population may also receive oppressive appearance-related messages from family members and peers, which could contribute to disordered eating attitudes and behaviors [83]. Thus, while the serial mediation pathway between GNAs, sexual racism, internalized racism, and disordered eating in the present study was statistically significant, it should be interpreted with caution. Future research should replicate and extend the current study to create a more comprehensive model by incorporating additional sociocultural factors, which may bolster the amount of variance explained.

### Limitations and future research

While the present study provides a novel understanding of queer Asian American men’s experiences with disordered eating, our findings should be interpreted with some drawbacks. First, our ability to make causal inferences and temporal effects was limited due to the present study’s cross-sectional design. Future research should thus utilize a longitudinal approach to examine the effects of GNAs on disordered eating via sexual racism and internalized racism over time. Specifically, to establish a temporal effect, studies may consider a three-wave longitudinal design to investigate GNA use and sex-seeking at baseline (T1), sexual racism six months later (T2), and internalized racism and disordered eating twelve months following baseline (T3). Furthermore, while the present study utilizes the term “geosocial networking apps” to refer to apps commonly used for dating and sexual activities to be consistent with previous research [84, 85], there may be geosocial networking apps that are not used for dating or sexual activities exclusively. Additionally, social justice-oriented scholarship has emphasized the importance of disaggregating data within the ethnically diverse Asian American diaspora and the culturally diverse sexual minority population [86–88]. Particularly, queer South Asian men have to contend with unique sexual racism experiences related to skin tone that queer East Asian men do not [89], indicating that queer Asian American men of different ethnicities may not internalize the same gendered racial stereotypes. Furthermore, a 2024 systematic review found that bisexual individuals experience unique sociocultural risk factors that render this community more vulnerable to disordered eating symptoms [5]. Future studies should thus investigate how sexual racism and internalized racism may manifest differently and affect disordered eating across subgroups of queer Asian American men. Moreover, studies have highlighted that queer men of color may engage in disordered eating to modify their muscularity [8, 90] and queer Asian American men may be particularly vulnerable to being dissatisfied with their muscularity levels [43, 53]. Considering how the disordered eating measure used in the present study did not target the specific dimension of muscularity, future research should utilize the Muscularity-Oriented Disordered Eating scale to capture this unique area of eating pathology among queer Asian American men [91]. Lastly, future research may continue building upon the culturally amended model of objectification theory by investigating whether sexual racism is associated with disordered eating through body surveillance and body shame [16].

Despite these limitations, the present study carries important implications for future social justice-informed prevention, clinical intervention, and advocacy efforts. In terms of clinical efforts, clinicians may consider integrating an intersectionality-grounded approach when working with queer Asian American men who experience disordered eating attitudes and behaviors. Furthermore, we encourage clinicians to consult the Awareness of Stereotype Internalization on Asian Narratives and Preventing Racism and Identity Distancing through Empowerment (ASIAN PRIDE) preventive intervention framework to support queer Asian American male clients in deconstructing harmful White supremacist ideologies [55]. For instance, building upon the ASIAN PRIDE framework’s emphasis on fostering ethnic pride and bicultural competence, we encourage clinicians to affirm their queer Asian American male clients’ racial identities and features. This approach may cultivate a sense of ethnic pride and reduce queer Asian American men’s desire to conform to Western appearance ideals, potentially diminishing the development of disordered eating. Aligning with the component of empowerment through social justice and individual and collective action [55], clinicians may foster liberation among their queer Asian American male clients who face sexual racism from GNAs by exploring strategies to resist White supremacy. These may include engaging in activism and advocacy, distancing from media as well as in-person spaces that center Whiteness, externalizing racism, and cultivating critical consciousness [92].

Our implications also extend beyond the therapy room. Considering the present study’s focus on GNAs, we recommend that prevention efforts begin in these online communities to target experiences of sexual racism and, subsequently, internalized racism, which may hinder the onset of disordered eating. We encourage public health preventionists to design initiatives that protect queer Asian American men from exposure to experiences of sexual racism in the first place. For instance, building upon the ASIAN PRIDE model’s emphasis on raising individual and collective awareness [55], GNAs may consider mandating educational advertisements on their platforms to inform users of the pervasiveness of sexual racism targeting queer Asian American men. Cultivating collective awareness may strengthen cross-racial solidarity across all users, minimizing the burden of resistance on queer Asian American men. Furthermore, GNAs may implement antiracist policies that integrate features that flag and remove profiles or interactions with racist language. Lastly, following the ASIAN PRIDE framework’s focus on deconstructing stereotypes and racist narratives that promote intra- and intergroup othering and inferiorization, we encourage the development of interventions across multiple levels (individual, systemic) [93]. Specifically, educational institutions and the media may work to systematically dismantle controlling images of queer Asian American men by replacing emasculating and fetishizing images of this community with affirming, positive, and empowering presentations [94].

## Conclusion

Our findings indicate that GNAs are an important factor associated with disordered eating among queer Asian American men through experiences of sexual racism and internalized racism. Future research could investigate protective factors that may reduce queer Asian American men’s risk for disordered eating. Furthermore, future disordered eating interventions should adopt an intersectionality-grounded and strengths-based approach to consider the role of experiences of racism in romantic and dating contexts and provide consciousness-raising strategies to combat internalized racism, which together may diminish queer Asian American men’s risk for disordered eating attitudes and behaviors.

## Data Availability

Data is available upon request from the first author.

## Abbreviation

GNA: Geosocial networking app
